# MAGNIFYING THE VOICE OF A COMMUNITY: ATTITUDES AND BELIEFS AMONG AFRICAN AMERICANS ABOUT DEMENTIA

**DOI:** 10.1093/geroni/igad104.0014

**Published:** 2023-12-21

**Authors:** Jabrina Simmons, Victoria Frager, Jia Broussard, Mikael Anne Greenwood-Hickman, Tyler Barrett, Deborah Barnes

**Affiliations:** Northeast Ohio Medical University, Rootstown, Ohio, United States; University of California San Francisco, San Francisco, California, United States; University of California, San Francisco, San Francisco, California, United States; Kaiser Permanente Washington Health Research Institute, Seattle, Washington, United States; Kaiser Permanente Washington Health Research Institute, Seattle, Washington, United States; University of California, San Francisco, San Francisco, California, United States

## Abstract

By 2060, the prevalence of Alzheimer’s disease and related dementias (ADRD) in the U.S. is predicted to more than double to 14 million. African Americans (A.A.) 65 and older are 2.5 times more likely to develop Alzheimer’s than same-aged White Americans. Furthermore, A.A. are twice as likely to be underdiagnosed, leaving the A.A. community underserved with respect to ADRD. To gain insight into the key drivers of this underdiagnosis, we performed a scoping review of research on attitudes and perceptions held within the A.A. community regarding ADRD, which may impact diagnosis and screening. We searched PubMed, CINAHL, Web of Science, and Black Studies Periodicals Database for studies from 2002 to 2022 using keywords: “Dementia” or “Alzheimer’s” and “African Americans,” “Ethnicity,” “Racial Groups,” “Blacks,” or “Ethnic and Racial Minorities,” and “Health Knowledge, Attitudes, Practice,” “Health Services Needs and Demand,” or “Perception.” We identified 318 studies; 49 met inclusion criteria after title and abstract review and were selected for full manuscript review. We discussed all included manuscripts and identified pertinent themes, noting potential method bias. An identified “knowledge gap” among A.A. about dementia, particularly a belief that dementia is part of normal aging or God’s will, was prominent. Other themes included mistrust of the medical community, a desire to maintain everyday family life, the need for culturally appropriate resources, and cultural expectations to provide care despite institutional support obstacles (John Henryism/Superwoman schema). Understanding differences in attitudes and beliefs toward dementia among African Americans may support developing effective strategies to address disparities.

